# Supplementary material for: Cognitive and Cultural Factors That Affect General Vaccination and COVID-19 Vaccination Attitudes
Source: Vaccines (Basel). 2022 Dec 30;11(1):94. doi: 10.3390/vaccines11010094 (PMC9865922; doi:10.3390/vaccines11010094)
Supplement: Supplementary file 1 [file vaccines-11-00094-s001.zip › vaccines-2036592-supplementary.pdf]

Covid-19 Vaccination Attitude Questions

The CDC website says that "COVID-19 vaccines are safe and very effective." Do you agree or disagree with the statement?

- Agree
- Somewhat agree
- Neither agree nor disagree
- Somewhat disagree
- Disagree

Please, explain: ____

Which statement best describes your attitude towards receiving Covid-19 vaccine?

- I already received one or both doses
- I want to get vaccinated as soon as I can
- I am considering getting vaccinated in the future, but don't feel ready yet
- I am not sure whether I want the vaccine
- I don't want the vaccine

Please, explain: ____

Which of the following is among your reasons for doubting getting the vaccine? (check all that apply):

- Not sure this vaccine is safe
- Not sure this vaccine is effective
- Not afraid of getting Covid
- Already had Covid*
- Scheduling an appointment is too difficult
- Getting to the vaccination site is not convenient
- This vaccine is conflicts with my religious beliefs
- Other (please specify)

*CDC recommends that you should be vaccinated regardless of whether you already had COVID-19

If you checked any boxes in the previous question, please explain your concerns

How may the following situation change your interest in receiving the vaccine? (the following answer options)

- Make me more interested in receiving the vaccine
- No effect
- Make me less interested in receiving the vaccine

Several family members and close friends got vaccinated. They did not have any side effects.
Several family members and close friends got vaccinated. Some had fever, chills, tiredness, and headache after the vaccine. These symptoms went away after a few days.
Many neighbors, colleagues, and members of your community got vaccinated. They are happy with the experience.
Your doctor or healthcare provider suggests that you should get vaccinated.
Doctors and nurses at the local hospital are excited to get the vaccine.
A community group you respect urges people to get vaccinated.
A faith leader you respect urges the congregation to get vaccinated.
A celebrity you admire campaigns to promote vaccination.
You read a CDC statement that Covid-19 vaccines are safe and very effective.
You read a detailed CDC explanation of how vaccines work. The explanation mentions scientific studies.
The case count of new COVID-19 cases spikes in communities where the percentage of vaccinated people is low.
A friend or relative suffers a severe COVID-19 related illness.

Where do you get your information about Covid vaccines? (check all that apply)

- Newspapers / news sites
- Health and wellness publications and websites
- TV
- Social media
- Web or mobile app forums (e.g., NextDoor)
- CDC
- Sources created by my local government
- Original scientific articles
- Your primary doctor or healthcare provider
- People I know
- Other (please explain)

How much do you trust the following sources to help you locate reliable information about Covid-19 vaccination?

- Friends and family
- Your primary doctor or healthcare provider
- Librarians in your local public library
- Librarians in a nearby hospital or medical school library
- Journalists
- Public health agencies

Demographic questions:

What is your age:

- 18-29
- 30-49
- 50-64
- 65+

What is your highest education level:

- High school or less
- Some college
- College graduate
- Postgraduate degree

Do you think of yourself as:

- Male
- Female
- Gender nonconforming, neither exclusively male nor female
- Additional gender category, or other
- Decline to answer

Do you identify as:

- American Indian or Alaska Native
- Asian
- Black or African American
- Hispanic or Latino
- Native Hawaiian or Other Pacific Islander
- White
- Other
- Decline to answerer

In what state do you live:

What best describes the area where you live?

- Urban
- Rural
- Suburban
- Frontier

Do you or anyone you live with go to work outside of the household?

- No
- Yes
- Not sure

Which of the following describes your occupation?

- Librarian
- Health or wellness professional (please explain)
- Biomedical researcher
- None

How important is religion in your life?

- Very important
- Somewhat important
- Not too important
- Not at all important
- Don’t know

How often do you attend religious services?

- At least once a week
- Once or twice a month / a few times a year
- Seldom / never
- Don’t know

What is your political party affiliation?

- Republican or leaning Republican
- No lean / Independent
- Democrat or leaning Democrat
- other

How would you describe your political ideology?

- Very conservative
- Conservative
- Moderate
- Liberal
- Very liberal
- Not sure

Do you watch cable news?

1. Yes
2. No

If you watch cable news, do you see it as a valid source of information about COVID-19 vaccines

1. Yes
2. No

Which cable news station do you watch most frequently?

1. CNN
2. Fox
3. MSNBC
4. Newsmax
5. One America News
6. Other

Information literacy survey

1. You want to find more information about making your immune system stronger. You type "boost immune system" into Google. From the results of that search, which website is likely the most reliable information source?
    a. www.health.harvard.edu
    b. www.healthline.com
    c. www.medlinx.com
    d. www.bbc.com

2. You go to the mercola.com website which features health news and articles. The website states that "The entire contents of this website are based upon the opinions of Dr. Mercola, unless otherwise noted." Does this mean the content has been reviewed by independent medical professionals (e.g., qualified doctors, nurses or other healthcare providers?)
    a. ___ Yes, definitely
    b. ___ No, not necessarily

3. Lisa has a toddler and is looking for a website with unbiased information about food to support her daughter's immune system. She finds 3 websites. Which of the sites is the best option for Lisa?
    a. Website A ends in .com. This website sells natural foods. There is no information about when the content was written or who wrote it.
    b. Website B ends in .info. The website content is written by a mother of 3 children and the information about food was last updated in March 2017.
    c. Website C ends in .gov. The website is maintained by a department of the U.S. federal government. The content has been checked by health professionals and the information about food was last updated in August 2020.

4. Which of the following authors would be the best qualified to write an article about the immune system?
    a. Maria Alonso, PhD, a food chemist
    b. Indira Acharya, NMD, a naturopath
    c. Sven Larsson, MA, a health blogger
    d. Pat Petersen, MD, an allergist

5. Which of the following sources' websites is most likely to provide accurate health information?
    a. An institute run by the U.S. federal government.
    b. A support group for patients living with a particular illness.
    c. A company selling medical devices.
    d. A social media company selling an app to connect patients with other patients.

6. The developers of a drink called BoostRx claim that their product increases effectiveness of the immune system. Which of the additional information below would provide the <u>strongest evidence</u> supporting this claim?

   a. Reviews by satisfied purchasers of the product.
   b. Links to published scientific articles written by one of the developers
   c. Links to published scientific studies of BoostRx, not conducted or sponsored by the developers.
   d. Advertising on BoostRx's website.

General Vaccination Attitude

Answer options: Agree, Somewhat Agree, Neither, Somewhat Disagree, Disagree

People who do not get vaccinated risk becoming very sick
Vaccines are effective in preventing diseases
I can get sick from vaccines
I am concerned that there may be something I don’t know about in vaccines

Trust survey

General trust

Generally speaking, would you say that most people can be trusted, or that you can't be too careful in dealing with people? Please rate your response on the scale from 1 to 5, where 1 means you can't be too careful and 5 means most people can be trusted.

Trust in health organizations

How much confidence do you have in the following institutions' ability to provide accurate, reliable health information? Please, rate your trust in them on the scale from 1 to 5, where 1 is you don't trust them at all and 5 is you trust them completely.

- Centers for Disease Control and Prevention, CDC
- National Institutes of Health, NIH
- Your primary doctor or healthcare provider
- A major university that conducts biomedical research
- A national health association, such as American Diabetes Association
- Food and Drug Administration, FDA
- A major pharmaceutical company

Trust in science

Do you agree or disagree with the following statements?

- Agree or mostly agree
- Neither agree nor disagree
- Disagree or mostly disagree

Science is the most reliable way of learning about the natural world.
Advances that are made in science are relevant to me and my community.
Scientific research with human subjects protects people who participate in it.
Science does more good than harm in the world.
I believe in science, but I don't trust scientists (because they may have other agendas).

SCIENTIFIC LITERACY SURVEY

1. *Many people who take multi-vitamins do not catch colds frequently. Thus, taking multi-vitamins prevents colds.* Is this a good scientific argument?
   a. *Yes*
   b. ***No***
2. *Scientists genetically engineered a strain of mice that lacked a certain gene. These mice could not reproduce. Scientists then re-introduced the gene back into the mice. Now the mice could reproduce. Thus, this gene is critical for mouse reproduction.* Is this a good scientific argument?
   a. ***Yes***
   b. *No*
3. *Nuplazid is a drug approved for treatment of Parkinson's disease. 34% of caregivers of people living with Alzheimer's believe that Nuplazid may also alleviate symptoms of Alzheimer's, because both diseases involve death of neurons. Thus, Nuplazid can be prescribed for Alzheimer's.* Is this a good scientific argument?
   a. *Yes*
   b. ***No***
4. *This year, there were 100,000 more cases of adolescent depression diagnosed in the US than last year. Thus, adolescent depression in the US is on the rise.* Is this a good scientific argument?
   a. Yes
   b. No
5. *A study randomly assigned 2,000 people who wanted to quit smoking into two groups. People in the first group participated in a weekly support group. People in the second group participated in weekly sessions of a cognitive behavioral therapy (CBT) smoking cessation program. At the end of 6 months, more people from the second group haven't smoked for a month. Thus, CBT is more effective for quitting smoking than participation in a support group*. Is this a good scientific argument?
   a. Yes
   b. No

<u>Take a look at the following descriptions of research studies. Evaluate the reasonableness of the conclusions.</u> Only use the information that is given below, without assuming anything else.

1. Researchers want to study how noise affects task performance. They randomly put participants into two groups. Females make up 35% of the first group and 75% of the second group. Participants in the first group complete a moderately difficult task in a quiet room. Participants in the other group do the same task in a noisy room. Researchers say that any differences in performance between the groups will be because of the noise. Based on this information only, do you see any other factors that may explain the difference?
   a. Yes
   b. No
      i. If yes, what is it?
2. Researchers want to study religious beliefs of students in U.S. universities. They send a survey to a random selection of 500 freshmen at a small private university in Tennessee. To the researchers, the findings represent religious beliefs of the U.S. university students. Based on this

information only, do you see any factors in the design that make you less confident about the researchers' interpretation of their findings?

   a. **Yes**
   b. No
      i. If yes, what is it?

3. To evaluate the effect of a new diet program, researchers compare weight loss between participants randomly assigned to treatment (diet) and control (no diet) groups, while controlling for average daily exercise and pre-diet weight. At the end, they ascribe differences between weight loss between the two groups to the program. Based on this information only, do you see any factors in the design that make you less confident about the researchers' interpretation of their findings?
   a. Yes
   b. **No**
      i. If yes, what is it?
4. Researchers compared the effectiveness of two arthritis medications. The first medication was tested in patients in a rural clinic in Midwest. Most participants were farmers who lived with their spouses and children. The second medication was tested in a nursing home in large city in the Northeast. Both groups had the same number of participants, who were following the same medication schedule. The group that received the first medication had better response, so the researchers concluded that the first medication is more effective. Based on this information only, do you see any factors in the design that make you less confident about the researchers' interpretation of their findings?
   a. **Yes**
   b. No
      i. If yes, what is it?
5. Researchers want to know whether stress contributes to sugar consumption. They conduct a large study in which, over a period of 5 years, they collect information on the diet and lifestyles of 100,000 people living in different regions and communities in the United States. In addition to diet and stress level questions and measures, researchers also collect information on family structure, work and leisure, income, education, and a number of other social and demographic factors. The researchers concluded that there is a connection between stress and sugar consumption. Based on this information only, do you see any factors in the design that make you less confident about the researchers' interpretation of their findings?
   a. Yes
   b. **No**
      i. If yes, what is it?
6. Researchers in a cancer clinic test a new drug in 12 patients with a rare cancer. None of the patients experience any dangerous side effects. The researchers concluded that the drug is safe. Based on this information only, do you see any factors in the design that make you less confident about the researchers' interpretation of their findings?
   a. **Yes**
   b. No
      i. If yes, what is it?

7. Scientists conducted a systematic review of 10 years of published studies in peer-reviewed journals that investigated the impact of CalmX on anxiety. The review included 214 studies, the methodological quality of which was judged by the systematic reviewers as ranging from "acceptable" to "high". Of these studies, two-thirds were funded by government institutions and one- third by the pharmaceutical industry. 186 studies found that CalmX reduced anxiety in the study participants, while 24 showed no effect. Based on this information, which of the following is most likely:
    a. CalmX is an effective drug for treating anxiety in most people.
    b. CalmX is not effective for treating anxiety. Some other factors, for which researchers did not control, interfered and made a difference. Researchers wrongly attributed the effect of that other factor to anxiety.
    c. CalmX is not effective for treating anxiety in most people. The designs of the studies were good, but, by pure chance, participants in the study had a very statistically unusual response.
    d. Any of the above is equally likely
